# The Role of Bronchoscopy and Chest Physiotherapy in Postoperative Patients With Acute Lung Atelectasis Due to Airway Mucus Plugging: A Case Series and Review of Entity

**DOI:** 10.7759/cureus.59324

**Published:** 2024-04-29

**Authors:** K K Athish, Guruprasad T J, Spurthy Padmanabha, Harshitha K R

**Affiliations:** 1 Internal Medicine, Sri Devaraj Urs Academy of Higher Education and Research, Kolar, IND; 2 Respiratory Medicine, Sri Devaraj Urs Medical College, Kolar, IND; 3 Pulmonology, Sri Devaraj Urs Academy of Higher Education and Research, Kolar, IND

**Keywords:** complication of mechanical ventilation, post abdominal surgery, postoperative pulmonary complications, acute lung collapse, pulmonary rehabilitation and medicine, chest physiotherapy, flexible bronchoscopy, lung atelectasis, postoperative lung collapse

## Abstract

Mechanical ventilation and endotracheal intubation can cause airway damage and inflammation resulting in excessive mucus secretions, thereby increasing the risk of respiratory failure post extubation. An abundance of secretions may result in bronchial obstruction and lung collapse distant from the site of obstruction. If lung collapse is diagnosed, extra support, including oxygen and, rarely, reintubation, can be necessary. The combination of chest wall percussion and vibrations, patient positioning to facilitate mucus drainage, coughing, and breathing exercises was the chest physiotherapy method employed for airway clearance in this study. Since the late 20th century, pulmonary rehabilitation strategies have been a standard aspect of care to prevent lung collapse in postoperative cases. Bronchoscopic aspiration and lavage are the common techniques used to remove retained secretions or mucus plugs. Large-volume saline instillation in aliquots and repeated suctioning are required during the procedure. Thus, the current case series emphasizes the role of bronchoscopy and pulmonary rehabilitation in the management of acute lung atelectasis during the postoperative period.

## Introduction

Flexible bronchoscopy (FB) has become increasingly important in a pulmonologist's daily practice since its introduction in the late 1800s. Its portability and versatility allow therapeutic interventions in addition to diagnosis, especially in critically ill patients and intensive care unit (ICU) settings. In this case series, we briefly discuss the postoperative cases requiring bronchoscopy for acute lung collapse with respiratory failure. Patients undergoing emergency abdominal surgery often experience postoperative pulmonary complications such as subsegmental/segmental/lobar atelectasis leading to respiratory failure, which increases the duration of hospital stay and the risk of mortality [1]. All the cases in this series developed respiratory failure, for which bronchoscopic toileting, followed by chest physiotherapy, was performed. Following the intervention, an improvement in oxygenation, general condition, and the resolution of atelectasis on the chest radiograph was noted.

Objective

The objective of this study was to know the role of bronchoscopy and pulmonary rehabilitation in improving lung expansion and oxygenation in patients with acute lung collapse secondary to airway mucus plugging among postoperative cases in the intensive care unit.

## Case presentation

Case 1

**Figure 1:**
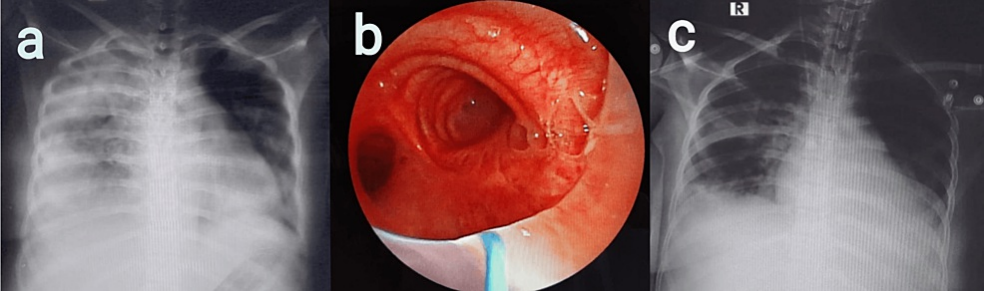
a) Chest radiograph revealed white-out lung on the right side, b) bronchoscopy revealed large mucus plugs obscuring the right mainstem bronchus and right upper lobe (RUL) bronchus, and c) post-bronchoscopy chest radiograph revealed lung expansion with a resolution of the collapse

A 26-year-old female presented to the emergency room (ER) with features suggestive of peritonitis secondary to hollow viscus perforation with septicemia and underwent exploratory laparotomy with peritoneal lavage and ileal anastomosis. Until postoperative day (POD) 5, the patient was hemodynamically stable, with minimal oxygen support. The patient was later shifted to the ICU, where mechanical ventilation was initiated because of a progressive drop in oxygen saturation. On examination, decreased breath sounds with signs of volume loss were noted on the right side. The chest radiograph revealed a white-out lung on the right side (Figure 1a). Suspecting acute right lung collapse secondary to mucus plugging, an emergency bronchoscopy was performed, and large mucus plugs obscuring the right mainstem bronchus and right upper lobe (RUL) bronchus (Figure 1b) were removed. Post bronchoscopy, a repeat chest radiograph revealed the resolution of the collapse with lung expansion (Figure 1c). Later, the patient was extubated given the improvement in oxygenation, general condition, and the radiological clearance of lung atelectasis. Chest physiotherapy was performed every four hours and consisted of five minutes of chest percussion, five minutes of postural drainage, and deep breathing to total lung capacity for three minutes with an incentive spirometer, which was continued until the discharge of the patient.

Case 2

**Figure 2:**
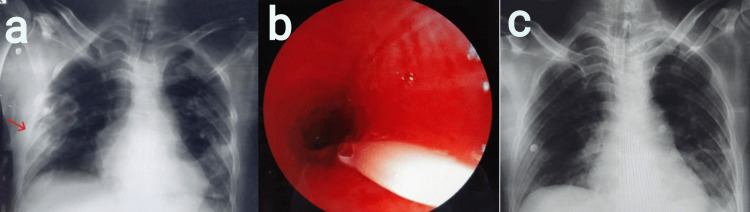
a) Chest radiograph revealed the segmental collapse of the right lower lobe, b) flexible bronchoscopy revealed mucus plug in the superior segment of the right lower lobe bronchus, and c) chest X-ray revealed the resolution of atelectasis following the removal of mucus plugs

A 46-year-old male was admitted to the ER with blunt trauma of the abdomen and peritonitis secondary to large gastric perforation following the alleged history of road traffic accident. The patient underwent exploratory laparotomy with primary suturing of gastric perforation with peritoneal lavage. Postoperatively, the patient was shifted to the ICU for ventilator support. On POD 2, the patient developed a drop in oxygen saturation, and the chest radiograph revealed a segmental collapse of the right lower lobe (Figure 2a). Flexible bronchoscopy revealed a mucus plug in the superior segment of the right lower lobe bronchus (Figure 2b). After removing mucus plugs, oxygenation and general condition improved. A resolution of atelectasis was noted on the chest radiograph post bronchoscopy (Figure 2c). Chest physiotherapy was performed every four hours and consisted of chest percussion and postural drainage each for five minutes. Post extubation, these techniques were combined with deep breathing to total lung capacity for three minutes with an incentive spirometer and continued until discharge.

Case 3 

**Figure 3:**
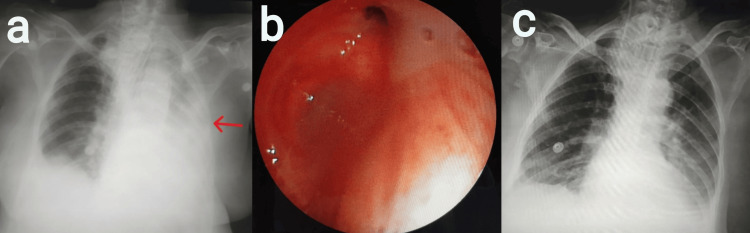
a) Chest radiograph revealed a left-sided white-out lung; b) bronchoscopy revealed mucus plugs blocking the left mainstem bronchus, left upper lobe (LUL) bronchus, and left lower lobe (LLL) bronchus; and c) post bronchial wash, a chest radiograph was performed, which showed the complete expansion of the left lung

A 62-year-old female patient, a known case of carcinoma of the ovary stage IIIC, post neoadjuvant chemotherapy, underwent interval debulking surgery (total abdominal hysterectomy + total omentectomy and parietal peritonectomy, cholecystectomy, and right diaphragmatic resection + distal pancreatic splenectomy + appendectomy + anterior peritoneal resection). Postoperatively, the patient was shifted to ICU for ventilator support. On POD 9, the patient was tracheostomized given the need for prolonged intubation. On POD 27, the patient had a drop in oxygen saturation. On examination, supra- and infraclavicular hollowing with reduced breath sounds was noted on the left side of the chest. A chest radiograph revealed a left-sided white-out lung (Figure 3a). Bedside ultrasonography (USG) of the thorax was performed to rule out pleural effusion. Emergency bronchoscopy was performed, which revealed mucus plugs blocking the left mainstem bronchus and left upper lobe (LUL) and left lower lobe (LLL) bronchi (Figure 3b). Post bronchial wash, the patient's oxygen saturation improved. A repeat chest radiograph showed the expansion of the left lung with a complete resolution of atelectasis (Figure 3c). Later chest physiotherapy was performed every four hours and consisted of chest percussion and postural drainage each for five minutes. After extubation, the above techniques were combined with deep breathing to total lung capacity for three minutes with an incentive spirometer. These methods were employed until discharge.

Case 4 

**Figure 4:**
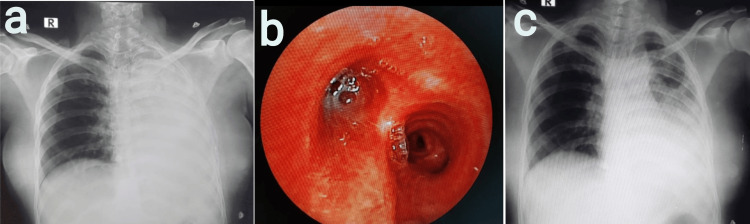
a) Chest radiograph revealed homogeneous opacities on the left side with a mediastinal shift to the left, b) bronchoscopy revealed left congested mucosa with large mucus plugs obscuring the left main bronchus, and c) chest radiograph revealed the expansion of the collapsed lung post bronchoscopy

A 36-year-old female with P2L1 underwent emergency lower-segment cesarean section (LSCS) under general anesthesia given a history of previous cesarean section with severe oligohydramnios in latent labor and short stature. The patient had a history of pulmonary tuberculosis 20 years ago. The patient complained of cough with expectoration on POD 1 and was treated with nebulization and antibiotics. On POD 2, the patient was shifted to the ICU because of a drop in oxygen saturation. On examination, fine inspiratory crackles, increased vocal fremitus, and increased vocal resonance were heard over the left mammary and interscapular area. Left lower lobe pneumonia with type 1 respiratory failure was diagnosed and treated appropriately. The ultrasonography of the thorax revealed no pleural effusion. Chest radiograph on POD 3 revealed homogeneous opacities on the left side with a mediastinal shift to the left suggestive of complete left lung collapse (Figure 4a). The patient underwent an emergency bronchoscopy, which revealed left inflamed mucosa with large mucus plugs obscuring the left main bronchus (Figure 4b). The left lower lobe bronchus also appeared stenosed with congested mucosa. Post procedure, bilateral breath sounds were of equal intensity. The patient maintained normal oxygen saturation, and the expansion of the collapsed lung was noted on the chest radiograph post bronchoscopy (Figure 4c). Contrast-enhanced computed tomography (CT) of the thorax revealed patchy areas of consolidation with few adjacent ground glass opacities in the left upper lobe, and calcified mediastinal and abdominal lymphadenopathy was noted. *Mycobacterium tuberculosis* was detected by cartridge-based nucleic acid amplification test (CBNAAT) in bronchoalveolar lavage (BAL) fluid. The patient was initiated on antitubercular therapy (ATT) and advised for chest physiotherapy and incentive spirometry. Chest physiotherapy was performed every four hours, which consisted of five minutes of chest percussion and five minutes of postural drainage. Deep breathing to total lung capacity for three minutes with an incentive spirometer was combined with chest physiotherapy following extubation.

## Discussion

As a standard of care, chest physiotherapy/pulmonary rehabilitation was provided regularly to all cases in the postoperative wards and the intensive care units. Of all four cases, three surgeries were performed on an emergency basis with one elective surgery, and all four cases have undergone abdominal surgeries. The age of cases was between 26 and 62 years. Three cases were found to have complete lung collapse, and one had a segmental collapse. In association with clinical deterioration (hypoxemia and decreased breath sounds), patients underwent a diagnostic roentgenogram to make a diagnosis of acute lung collapse. However, all four cases required mechanical intubation before therapeutic bronchoscopy.

Acute lobar atelectasis is defined as volume loss that occurs with characteristic radiological patterns such as bronchovascular crowding, the increased opacity of the affected lobe, rib space narrowing on the ipsilateral side, the compensatory hyperinflation of the unaffected side, mediastinal shift to the affected side, elevated diaphragm, hilar displacement, the loss of volume on ipsilateral hemithorax in massive collapse, and the loss of cardiac silhouette along with clinical deterioration [2]. According to a randomized trial by Edmark et al., a statistically significant difference in the size of lung atelectasis was noted between never-smokers and smokers (active and former smokers); however, it is still unclear if the study results are clinically relevant. Another novel discovery was the correlation between the American Society of Anesthesiologists (ASA) class and atelectasis, which was more significant than the correlation between smoking and atelectasis. This raises the dilemma of whether ASA class is the common denominator across several patient characteristics that contribute to the establishment and progression of atelectasis [2]. All the cases in this series were never smokers. Cases 1, 2, and 4 belong to ASA E, whereas case 3 belongs to ASA III.

The resorption of gas behind closed airways and a decreased respiratory muscle tone can cause atelectasis during anesthesia and postoperative periods [3]. Gas exchange and ventilation are frequently compromised throughout the surgery and the postoperative period. The development of atelectasis and shunt during anesthesia is a primary factor contributing to the reduced gas exchange, which is further compromised by an increased ventilation-perfusion (V/Q) mismatch. Postoperatively, an impaired gas exchange may be caused by residual anesthesia, discomfort, immobilization, and decreased sensitivity to hypoxemia and hypercarbia. Lower vital capacity and diaphragmatic function appear to be compromised by upper abdominal surgery by at least 50%. Postoperatively, dilated bowels, abdominal dressings, reduced forced vital capacity (FVC), and forced expired volume in one second (FEV1) will further encourage the intermittent and even continuous closure of airways during tidal breathing [4].

Mucus is a normal secretion from the tracheobronchial tree. Postoperative patients and ICU patients requiring prolonged intubation are at risk of acute lung collapse, commonly by endobronchial intubation or the blockage of the tube by secretions or blood. In the above conditions, the patient's natural process of mucus clearance by coughing will be compromised. This results in the accumulation and thickening of mucus within the airways leading to airway obstruction and the impairment of oxygen exchange. The mechanisms of hypoxemia are V/Q mismatch, hypoventilation, diffusion impairment, and shunts [5].

The critical clinical state of the patient makes the chest radiograph a preferred diagnostic modality compared to a computed tomography (CT) scan, although the former can not differentiate mucus plugging from other disease processes, which is possible with a CT scan [6].

Pulmonary rehabilitation or chest physiotherapy has been demonstrated to prevent or even mitigate respiratory illnesses such as atelectasis and pneumonia. Respiratory physiotherapy techniques help reduce pulmonary problems when used in conjunction with postoperative treatment [7]. Upon the onset of symptoms, the early initiation of chest physiotherapy in the form of conventional pulmonary rehabilitation, the use of oscillatory devices, manually assisted cough, mechanical insufflation-exsufflation, and positive expiratory pressure (PEP) might prolong or avoid the need for an invasive procedure. However, bronchoscopic intervention remains the primary modality of treatment for acute lobar or segmental atelectasis due to mucus plugging [8]. The effectiveness of bronchoscopy and chest physical therapy, postural drainage, percussion, suctioning, and lung expansion in treating atelectasis is comparable [9].

Zeng et al. found that patients with atelectasis who received both chest physiotherapy and kinetic therapy (KT) fared better radiographically and in terms of oxygenation indices than those who only received KT [10]. A comparative study by Stiller et al. revealed a much higher rate of the resolution of acute lobar atelectasis after a single therapeutic intervention with positioning and vibrations along with hyperinflation and suction than patients who received a combination of hyperinflation and suction alone. These results were found to be statistically significant at the end of the six hours of intensive treatment, whereas the outcomes compared at the end of the first 24 and 48 hours were not found to be statistically significant [11]. It is advised that severely ill patients with atelectasis receive chest physiotherapy at least every two hours [12]. In the management of acute lobar atelectasis, Marini et al. carried out a small randomized control trial comparing flexible fiberoptic bronchoscopy (FFB) with bronchoalveolar lavage (BAL) to chest physiotherapy, of which 14 patients underwent emergency bronchoscopies, with chest physiotherapy administered every four hours over 48 hours. An additional 17 patients were randomized to receive just pulmonary rehabilitation every four hours, with an FFB performed every 24 hours if the atelectasis persisted. In contrast to their endeavors, there was no statistically significant variation in the resolution rates among the two groups. After 24 hours, around 80% of the volume loss was restored in both groups. The authors believed that the reason for this discrepancy was that their inclusion criteria only included patients with complete or near-complete lung collapse [9].

Snow and Lucas conducted a prospective study to look at the results and the morbidity of 51 patients who underwent surgical intensive care after bronchoscopy procedures. Nearly two-thirds of the cases had lobar collapse as the main procedural indication. Compared to other indications such as persistent pulmonary infiltrates and suspected aspiration, they noticed a considerable resolution in cases with lobar collapse post bronchoscopy. They did not, however, compare bronchoscopy with conservative treatment [13]. Most of the literature states that bronchoscopic toileting is more effective in treating patients who exhibit lobar atelectasis without an air bronchogram on chest radiography [13]. In our case series, we have performed bronchoscopic toileting in patients who had segmental/subsegmental atelectasis and/or the presence of an air bronchogram. Following bronchoscopic toileting, we have observed the improvement in oxygenation and chest radiography of these patients with segmental/subsegmental atelectasis and/or the presence of an air bronchogram. In cohort analysis between patients receiving bronchoscopy and conservative measures only for lung atelectasis, Toolsie et al. reported statistically significant differences among the patient group who underwent bronchoscopy for the clearance of lung collapse. The ability to directly examine the airway and the possibility of obtaining bronchoalveolar lavage (BAL) or tissue specimens for conclusive diagnosis are the two most important advantages of the bronchoscopy technique [14].

As hypoxemia and respiratory failure are clinical indications for the procedure, flexible bronchoscopy should not be delayed. The indications for FFB lavage are lobar collapse not resolving with aggressive pulmonary rehabilitation and total lung collapse. On the contrary, the existence of an air bronchogram in a chest radiograph (suggests patent airway) or prolonged left lower lobe collapse following abdominal or thoracic surgery has demonstrated the reduced effectiveness of FFB in the management of atelectasis [8]. As per Menditto et al., the retention of secretions in the lungs and atelectasis were the indications for emergency flexible bronchoscopy (FB) in the intensive care unit [15].

Due to the inherent limitations of a case series design, this study cannot establish definitive conclusions or causal relationships. The small sample size (n = 4) restricts the generalizability of our findings to a broader population. Additionally, incorporating standardized preoperative pulmonary function assessments would allow for a more robust evaluation of treatment efficacy. Future prospective studies with a larger and more diverse patient cohort are warranted to investigate the potential benefits of chest physiotherapy. By employing a comparative design, such studies could directly compare the outcomes of chest physiotherapy alone to the combined approach with bronchoscopic toileting, providing valuable insights into the potential synergistic effects of these interventions.

## Conclusions

The strict provision of pulmonary rehabilitation reduces the incidence of postoperative pulmonary complications including acute subsegmental, segmental, and lobar atelectasis. This case series underscores the need for bronchoscopy to manage patients with respiratory failure due to acute lung atelectasis secondary to the mucus plugging of the central and distal airways.
